# Validation of Autism Spectrum Quotient Adult Version in an Australian Sample

**DOI:** 10.1155/2013/984205

**Published:** 2013-05-12

**Authors:** J. Broadbent, I. Galic, M. A. Stokes

**Affiliations:** School of Psychology, Deakin University, 221 Burwood Highway, Burwood, VIC 3125, Australia

## Abstract

The Autism Spectrum Quotient is used to assess autistic spectrum traits in intellectually competent adults in both the general population and the autism spectrum community. While the autism spectrum Quotient has been validated in several different cultures, to date no study has assessed the psychometrics of the Autism Spectrum Quotient on an Australian population. The purpose of this study was to assess the psychometrics of the autism spectrum Quotient in an Australian sample of both typically developing individuals (*n* = 128) and individuals with autism spectrum disorder (*n* = 104). The results revealed that the internal consistency and the test-retest reliability were satisfactory; individuals with autism spectrum disorder scored higher on total Autism Spectrum Quotient score and its subscales than typically developing individuals; however, gender differences were not apparent on total score. Possible cultural differences may explain some of the psychometric variations found. The results of this analysis revealed that the Autism Spectrum Quotient was a reliable instrument for investigating variation in autistic symptomology in both typically developing and Autism Spectrum Disorders populations within an Australian population.

## 1. Introduction

Autism spectrum disorders (ASD) are a group of disorders marked by impairments in social communication and repetitive behaviors [1]. While impairments in these areas must be present in order to meet the specific criteria for a positive diagnosis [2], there is growing evidence that severity of symptomology occurs along a continuum which ranges from severely impaired to low impairment not able to meet diagnostic criteria [3]. This suggests that it may also be possible for autistic traits to be normally distributed within the general population, where typically developing individuals display autistic traits that vary in both degree of severity and number [4].

A common measure of autistic traits is the Autism Spectrum Quotient (AQ), designed by Baron-Cohen et al. [5] to assess Autistic Spectrum traits in intellectually competent adults in both the general population and the Autism Spectrum community. It is a 50-item questionnaire designed to assess five different areas of functioning: social skills, attention switching, attention to detail, communication, and imagination. The Total AQ score, which has a minimum total score of 0 and a maximum overall score of 50, has been used to differentiate individuals with an ASD from typically developing (TD) individuals in adults [5], adolescents [6], and children [7]. While the AQ purports not to be diagnostic, scores are thought to screen individuals with a potential diagnosis of ASD. Assessments of the psychometrics of the AQ have established a differentiation cut-off score of 32 or above, capturing 80% of individuals with ASD, but with a 2% false positive rate that leads to many TD individuals being diagnosis incorrectly [5].

The AQ total score is continuously distributed in both an ASD and general population, with several studies reporting high internal reliability above 0.7 [5, 7–9]. In the general population the AQ has also been shown to be sensitive to differences in gender, with males in the general population scoring significantly higher than females [5–7, 9–11]. In addition Auyeung et al. [7] found that male children scored higher (displaying greater Autistic tendencies) on the subscales of Social Skills, Communication, and Imagination. Findings have also shown the AQ has no bias towards any particular age group [3, 6, 12]. These norms have been replicated in several UK samples [5, 10, 13], a Dutch sample [8], a Scottish sample [11], a French-Canadian sample [14] a US sample [15], and several nonwestern samples [9, 16, 17].

No published studies to date have used the AQ with an Australian sample of individuals diagnosed with ASD. Given that the cultural variation of an Australian sample may affect item interpretation and the growing popularity of the AQ as a measure of autistic symptomology, it is important that the psychometrics be empirically demonstrated in an Australian sample. Based on the aforementioned studies, it was predicted that (a) the AQ will be continuously distributed within the general population; (b) the AQ will be able to differentiate individuals with ASD from TD individuals; (c) males will score higher on the AQ and its subscales than females; and (d) AQ scores will not be related to age.

## 2. Method

### 2.1. Participants

Participants were recruited from an ongoing and completed study if they were aged between 16 and 65 years. A total of 233 returned AQ questionnaires. See Table 1 for description of age. 

Participants in the TD group (*n* = 129) were recruited by word of mouth through acquaintances of the authors and were part of a larger study. All participants in the ASD group (*n* = 104) had a diagnosis in line with DSM-5 (which no longer distinguishes subtypes of ASD). These participants were recruited through autism Victoria support networks and autism practitioners in Melbourne, Australia, and were only included in the sample if their diagnosing practitioner had also tested their IQ, and the result was above 70. No other IQ details were requested. The participants with ASD were also part of a larger study. It was assumed that if TD individuals did not have a diagnosis that included an intellectual disability, they would fulfill this criterion, and thus be able to participate.

An independent samples *t*-test revealed that the ASD group was significantly older than the TD group, *t*_(156.08)_ = 3.77, *P* > .05, *d* = .60. Consequently, age was covaried in all analyses where covariance was appropriate and also where age itself was not an IV or DV. Further, Chi-square analysis also indicated there was a significant difference between the number of males and females in each group, *χ* ^2^_(1)_ = 9.42, *P* < 0.01.

### 2.2. Materials

#### 2.2.1. Demographic Questionnaire

A set of demographic questions asked participants their gender and their age in years. In addition, participants with ASD were required to give full details of their diagnosis and the diagnosing practitioner.

#### 2.2.2. The Autism Spectrum Quotient (AQ)

The AQ was developed by Baron-Cohen and colleagues in 2001 [5]. The AQ is a brief 50-item self-administered questionnaire, which is designed to assess Autistic Spectrum traits in the general population. The AQ is divided into five different areas of functioning related to autistic traits: social skills, attention switching, attention to detail, communication, and imagination. See Baron-Cohen et al. [5] for scoring.

### 2.3. Procedure

Approval from the Deakin University Human Research Ethics Committee (DUHREC; EEC013-2007 & EC00213-2009) was obtained for this study. Participants were asked to self-administer the questionnaires in their own time and preferably alone, either online or in hard copy (returned by mail). Participants were informed that it would take 20 minutes to complete the questionnaire.

## 3. Results

### 3.1. Data Screening

Missing data points constituted 0.2% of the data overall and were dealt with by using expectation maximisation-based imputation. Subsequent scans of the complete data, using a *z* score criteria of ±3.29, revealed no univariate outliers.

The Total AQ score and its subscales were assessed for normality through an examination of absolute skew and kurtosis scores for each variable; the data was not found to deviate from normality.

### 3.2. Scale Reliability

Internal reliability consistency coefficients using Cronbach's alpha were derived for the TD and the ASD groups (see Table 2). Internal consistencies for the separate ASD and TD groups ranged from unacceptable to good. Given the low Cronbach's alpha values for several subscales (for TDs), the interitem correlation matrix was checked for potential sources of low internal consistency. It was clear from this matrix that the items do not correlate well and in some instances among TD subjects there were small, negative correlations, resulting in a lower Cronbach's alpha. However, these findings are similar to those found by others [5, 9, 10, 15], with similarly low scores for TD individuals on attention switching, communication, and imagination as found by Hurst et al. [15] but not Austin [10], Baron-cohen et al. [5], or Wakabayashi et al. [9].

### 3.3. Test-Retest Reliability

Twenty-seven participants (17 TD and 10 ASD participants) completed a second copy of the AQ to examine test-retest reliability. The interval between completing the AQ in time one and time two spanned 6 to 12 months.  Table 3 shows that the correlation between time one and time two scores on the AQ was high.

### 3.4. Distribution of AQ Total Scores

**Figure 1:**
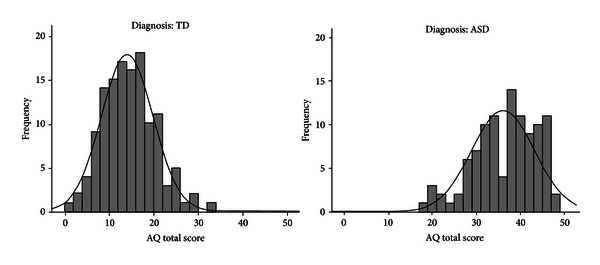
Frequency of Total AQ score by diagnosis.

The total AQ scores were analysed separately for both ASD and the TD groups and were found to be normally distributed, Kolmogorov-Smirnov *Z* = 1.04, *P* > .05 and *Z* = 0.75, *P* > .05, respectively.  Figure 1 reveals that TD individuals had a lower Total AQ score of *M* = 14.05 (SD = 5.80), compared to ASD individuals' Total AQ score of *M* = 36.04 (SD = 7.13); this difference was significant, *t*_(197.06)_ = 25.42, *P* < .001, and *d* = 3.62. 

### 3.5. Distribution of AQ Subscales

**Figure 2:**
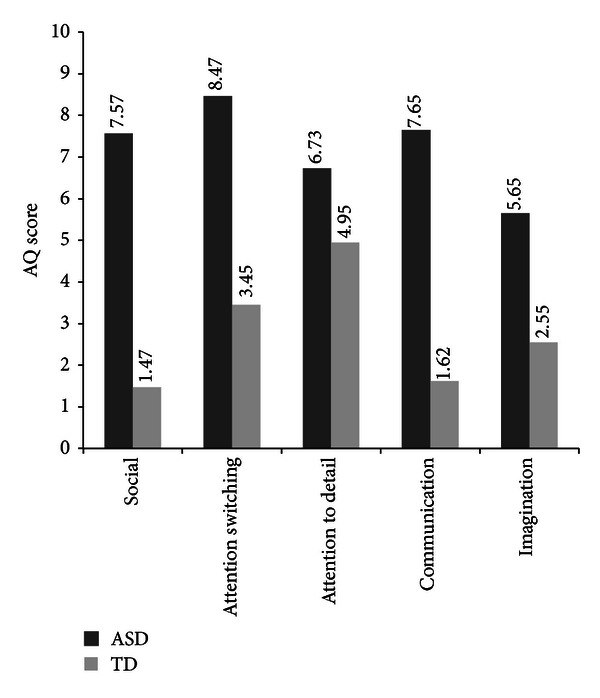
Distribution of scores for the AQ subscales social skills, attention switching, attention to Detail, communication, and imagination.

As Total AQ score was significantly different between TD and ASD participants, it would be expected that the subscales of the AQ should also be able to differentiate diagnosis.  Figure 2 shows the mean scores of the subscales for the ASD and TD groups. In order to undertake analysis of the subscales, age was included as a covariate (CV), removing variance due to age differences between the groups. A test of homogeneity of regression was undertaken, and results were satisfactory; age was deemed sufficiently reliable for covariance analysis. The differences on the subscales of the AQ between TD and ASD were found to be significant even after controlling for differences in age, with individuals with ASD scoring higher on all scales (Table 4).

### 3.6. Cut-Off Scores of AQ Total

**Figure 3:**
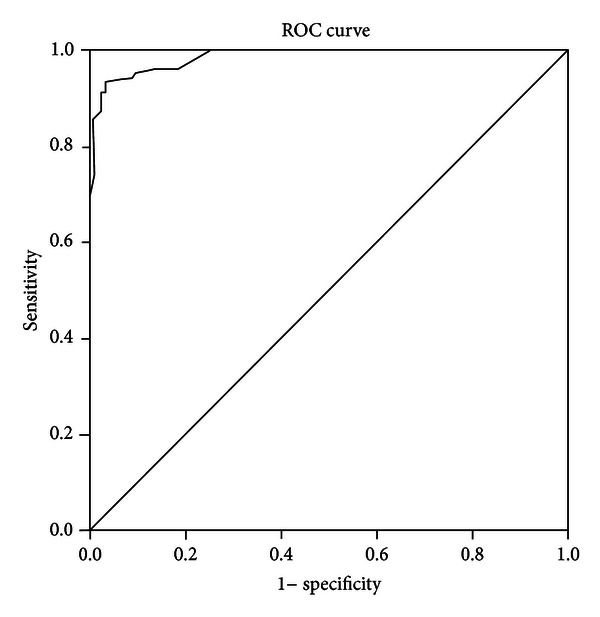
ROC curve of the sensitivity and specificity of the AQ-Adult score. Area under the curve = .99.

Baron-Cohen et al. [5] recommended a cut-off point for Total AQ score of 32, which they found should capture 80% of those diagnosed with ASD, but with a 2% false positive rate.  Table 5 shows sensitivity (proportion of individuals with ASD who are correctly identified as such) and specificity (the proportion of typically developing individuals who are correctly identified as such) values for a range of potential cutoffs, and Figure 3 gives the ROC curve; the area under the curve was .99. As it is believed that about 1% of the population is persons with ASD [18], then a false positive rate greater than this would be unacceptable. Thus, a cutoff at 29 appears to be the best in the current sample, which gives a 14.4% false negative rate and a less than 1% false positive rate. Note we could not determine the actual rate of false positives in this group.

### 3.7. Gender and AQ Total and Subscale Scores

**Figure 4:**
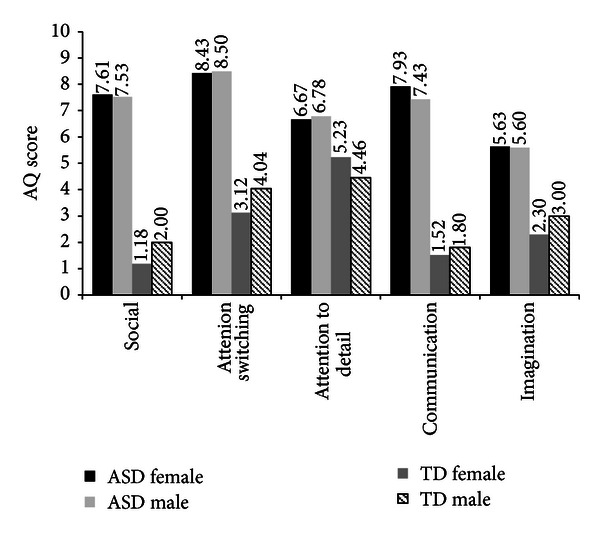
Mean scores on the AQ subscale scores for males and females of TD and ASD groups.

**Figure 5:**
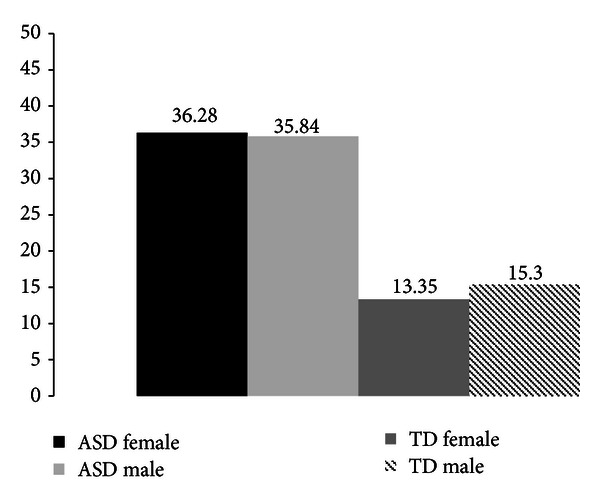
Mean scores on the total AQ for males and females of TD and ASD groups.

Past research has shown there to be gender differences in Total AQ score, with males scoring higher than females in Total AQ [5, 6, 8, 10] and on the subscales such as social skills, communication, and imagination [8]. This is found to be especially so in general population cohorts. Following on from this, the mean scores of Total AQ scores by gender and diagnosis were analysed (see Figures 4 and 5). 

The mean score comparisons for gender and AQ Total score show that TD males scored slightly higher than TD females and that ASD males scored marginally lower than females on Total AQ score. However, an ANCOVA revealed that there was no significant difference for gender by diagnosis on any of the scales of the AQ (see Table 6). Note Cohen's *d* has been calculated for comparisons with other studies.

### 3.8. Age and AQ Total and Subscale Scores

Studies have commented that there is no relationship between AQ total score and age of participants [7, 17]. In order to establish whether the current dataset provides support for these findings, age and Total AQ scores were correlated (see Table 7). 

The results of the Pearson's correlation show that within the TD group, *age* had a moderately positive relationship with Total AQ, Social Skills, Communication and Imagination, with a weak negative correlation with *Attention to Detail*. Deficits in these areas increased with age except the subscale Attention to Detail. For the ASD group, age was significantly correlated with Imaginationscores, indicating for this cohort, deficits in imagination increased with age.

## 4. Discussion

This study psychometrically evaluated Baron-Cohen et al.'s [5] adult version of the AQ with an Australian population. No other studies have validated the AQ with an Australian sample of individuals diagnosed with ASD or with a typically developing population. 

While the AQ subscales (with the exception of *Attention Switching*, *Cα* = .52) exhibited good psychometric properties for the ASD group, for which the scale is designed [5], reliability estimates tended to be lower for TDs than ASDs. In fact, two subscales performed poorly in the TD group: Communication (.49) and Imagination (.40). These findings are similar to those found by Hurst et al. [15] but were not replicated in studies from the UK [5, 10].

There are several possible explanations for poorer alphas across most subscales for TD. First, it is possible that the small number of items in each subscale contributed to the lower alpha levels, as it is known that as the number of items decreases, Cronbach's alpha tends to decrease [19]. Second, the fact that Cronbach's alpha values in the present study are lower than those reported in UK samples [5, 10] suggests possible cultural differences in how participants responded to items. Other research outside of the UK has also found low-to-moderate internal consistencies [15], which supports a conclusion that some of the AQ subscales may be sensitive to culture. However, given the paucity of studies validating the AQ conducted outside of the UK, additional research is needed to assess the possibility of cultural sensitivity in the AQ. Formal comparisons of item functioning, using item response modeling or measurement invariance tests, would help to clarify this issue. Until such matters are resolved, it is recommended that analyses using the AQ subscales with Australian samples should be interpreted cautiously. 

Overall, however, the AQ displayed good test-retest reliability indicating the construct is stable over time. The majority of the reliability findings support the structure of the questionnaire, indicating AQ and its subscales consistently measure the same construct. These findings are mostly in line with, and in some cases somewhat better than other studies [5, 10, 15].

As found in previous studies [5, 8, 9] AQ scores in the TD and ASD groups had an approximately normal distribution. However, it must be noted that the ASD AQ total score distribution does not appear entirely normal, with a few cases toward the negative tail and a few holes in the central body. Regardless, these results suggest that the AQ reflects the degree of autistic symptomatology in accordance with the notion that these traits are part of a broader phenotype, on which characteristics lie along a continuum.

The ASD group scored significantly higher on Total AQ than the TD group. Further, this significant difference was seen on all the subscales of the AQ, with ASD individuals scoring significantly higher on each subscale than TD. This is in line with Baron-Cohen et al.'s [5] original findings and suggests that the AQ has acceptable discriminative validity since the AQ is designed to measure autistic symptomatology and has demonstrated doing so in the current study.

While the AQ purports not to be diagnostic, scores are thought to screen individuals with a potential diagnosis of ASD. The original cutoff of 32 [5] has been suggested to correctly identify individuals with autistic traits; this has been supported by Baron-Cohen et al.'s [6] findings in the adolescent version of the AQ. Albeit, this threshold value has been challenged by [7, 10, 14, 20] who reported acceptable cutoff scores of 30, 30, 26, and 22, respectively. The present study found that cutoffs of 29 showed both high sensitivity and high specificity, and resulted in the correct classification of the greatest percentage of participants. Most importantly, a cut of 29 allows for only 1% false positive rate. This is most closely in line with [7, 10], cutoff of 30. 

It must be kept in mind that where individuals score high on the AQ, this does not designate a probable diagnosis of ASD. Further, it is worthwhile noting that Baron-Cohen et al.'s [5] acceptable false positive rate of 2% still means 2% of all TD individuals will be classified as having a probable diagnosis of ASD. As less than 1% of all people have an ASD, this means there would be twice as many with a probably diagnosis of ASD as there should be. This, it could be argued, is an unacceptably high rate.

Typically developing males have been found to have consistently higher AQ total scores compared to TD females [5–11, 14], with studies reporting that these gender differences also extend to the subscales of the AQ [10, 21]. However, group differences relating to gender in the present study were not as expected. The present study failed to find an effect of gender by diagnosis on either AQ total or subscale scores. It is possible, however, that the gender differences may have reached significance had the sample size (*n* = 233) or effect sizes (*d* = .16–.23) been larger. Previous studies all had sample sizes ranging between 50 and 1,261 participants. On the other hand, Hurst et al. [15] also found no gender difference with a sample size of 1,005 participants. In studies where gender had been found to have an effect [5–11] sizes ranged from *d* = .23–.97, with the smallest effect size matching the current study's largest. It is questionable though how clinically important this difference may be, as the effect size observed here only accounted for.7%–1.5% of all variance. 

Of the studies to explore the effect of age on AQ total score, all asserted that no significant age effect on AQ total score was found [6, 7, 17]. In contradiction, the present study observed some significant age effects on Total AQ, and the subscales Social Skills, Attention to detail, Communication, and Imagination for some groups. These findings suggest some sensitivity to age exists within the AQ, which argues against the claim that the test is not influenced by age. 

The study had several limitations. As mentioned previously in this discussion, the sample size in this study was small and thus findings should be interpreted with care. Further, as the AQ requires a self-report of behaviours, thoughts, and feelings, there is some possibility of response bias. This may be particularly true for the ASD group, who may have poor insight into their own behaviour. Despite this, the AQ combats response bias by (a) wording items with an equal number of positive and negative response sets and (b) including items in the social and communication domains that ask for a person's preference, rather than asking them to make their own judgment about their behavior; therefore, it has been argued bypassing any limitations in insight [5].

Another possible limitation is that the sampling procedures used in the current study were not as rigorous as those employed by Baron-Cohen et al. [5]. For example, the current study did not test IQ, and there were significant differences in age between the TD and ASD groups. Thus Baron-Cohen et al.'s more restrictive sample, particularly in regard to the ASD group, may account for the issues found with internal consistency of the AQ herein, and a lower cut-off score distinguishing between individuals with an ASD and TD individuals was found in the present study. However, if the AQ is to be used as either a screening instrument within Australia, or as a research instrument to validate the stated diagnoses of research participants, it needs to be able to cope with IQ differences between TD and ASD groups.

In conclusion, the present study suggests that despite some psychometric differences found, the AQ is a reliable instrument for investigating variation in autistic symptomatology in the general and ASD Australian populations. It displays traits along a continuous distribution with mostly acceptable internal consistency and test-retest reliability, which gives support to the structure of the test. Further, the AQ appears to be useful for distinguishing individuals with high levels of autistic traits from TD individuals and, although not developed as a diagnostic tool, shows merit as a screening tool.

## Figures and Tables

**Table 1:** Mean age for ASD and TD participants.

	Mean age in years	SD
TD total (*n* = 129)	27.28	8.06
TD female (*n* = 83)	26.22	6.94
TD male (*n* = 46)	29.20	9.54
ASD total (*n* = 104)	33.12	14.04
ASD female (*n* = 46)	32.52	13.85
ASD male (*n* = 58)	22.60	14.28

**Table 2:** Internal consistencies (Cronbach's alpha) for all subscales of the AQ for TD and ASD groups.

Scale	TD (*n* = 128)	ASD (*n* = 104)
Total (50 items)	.75	.84
Social Skills (10 items)	.73	.69
Attention Switching (10 items)	.56	.52
Attention to Detail (10 items)	.65	.68
Communication (10 items)	.49	.64
Imagination (10 items)	.40	.69

**Table 3:** Independent samples *t*-tests, means, standard deviations, and bivariate correlation for the test-retest reliability of the AQ and its subscales for TD (*n* = 17) and ASD (*n* = 10) participants.

AQ scales	*M*_*T*1_	SD_*T*1_	*M*_*T*2_	SD_*T*2_	*r*
Total	21.74	14.56	21.59	14.41	.95**
Social Skills	4.30	4.18	3.81	3.90	.79**
Attention Switch	5.30	3.56	5.15	3.48	.96**
Attention Detail	5.04	2.31	5.00	2.29	.79**
Communication	3.74	3.78	3.70	3.61	.97**
Imagination	4.22	3.09	3.93	2.88	.75**

*Note. T1* = time one, *T2* = time two.

**P < .001.

**Table 4:** ANCOVAs comparing TD and ASD scores on the subscales of the AQ controlling for age.

	*F*	df_1_	df_2_	*P*	*η* ^2^
Social skills	479.57	1	230	<.001	.68
Attention switching	393.81	1	230	<.001	.63
Attention to detail	36.80	1	230	<.001	.14
Communication	602.18	1	230	<.001	.72
Imagination	108.54	1	230	<.001	.32

**Table 5:** Detailed report of diagnostic statistics for the Autism Spectrum Quotient (AQ) and discriminative ability of AQ cut-off scores.

Cut-off point	Sensitivity (%)	Specificity (%)
>1	100.0	0
>2	100.0	0.8
>3	100.0	1.6
>4	100.0	2.3
>5	100.0	3.1
>6	100.0	5.4
>7	100.0	9.3
>8	100.0	12.4
>9	100.0	16.3
>10	100.0	23.3
>11	100.0	28.7
>12	100.0	34.9
>13	100.0	42.6
>14	100.0	48.1
>15	100.0	54.3
>16	100.0	60.5
>17	100.0	69.8
>18	100.0	74.4
>19	99.0	77.5
>20	96.2	82.2
>21	96.2	86.8
>22	95.2	90.7
>23	94.2	91.5
>24	94.2	93.0
>25	93.3	96.9
>26	91.3	96.9
>27	91.3	97.7
>28	87.5	97.7
**>29**	85.6	99.2
>30	82.7	99.2
>31	78.8	99.2
**>32**	75.0	99.2
>33	69.2	100.0
>34	64.4	100.0
>35	58.7	100.0
>36	55.8	100.0
>37	54.8	100.0
>38	45.2	100.0
>39	41.3	100.0
>40	37.5	100.0
>41	30.8	100.0
>42	26.0	100.0
>43	22.1	100.0
>44	17.3	100.0
>45	12.5	100.0
>46	4.8	100.0
>47	1.9	100.0
>48	1.0	100.0
>49	0.0	100.0
>50	0.0	100.0

**Table 6:** ANCOVAs comparing males and females with diagnostic group on scores on the subscales of the AQ controlling for age.

	*F*	df_1_	df_2_	*P*	*d*
AQ Total	1.65	3	228	.20	.17
Social Skills	2.33	3	228	.13	.20
Attention Switching	2.96	3	228	.09	.23
Attention to Detail	1.95	3	228	.16	.18
Communication	2.48	3	228	.12	.21
Imagination	1.46	3	228	.23	.16

**Table 7:** Bivariate correlations of age and AQ subscores for TD and ASD groups.

	TD (*n* = 129)	ASD (*n* = 104)
Total AQ	.20*	.19
Social Skills	.46**	.18
Attention Switching	−.03	.16
Attention to Detail	−.17*	−.01
Communication	.28**	.05
Imagination	.19*	.26**

*Two-way *P* < .05;  **two-way *P* < .01.
